# Multimodal Sensing Strategy Using pH Dependent Fluorescence Switchable System

**DOI:** 10.1038/srep39452

**Published:** 2016-12-22

**Authors:** A. Muthurasu, V. Ganesh

**Affiliations:** 1 Electrodics and Electrocatalysis (EEC) Division, CSIR – Central Electrochemical Research Institute (CSIR – CECRI), Karaikudi – 630003, Tamilnadu, India; 2 Academy of Scientific and Innovative Research (AcSIR), New Delhi – 110025, India

## Abstract

Biomolecules assisted preparation of fluorescent gold nanoparticles (FL–Au NPs) has been reported in this work using *glucose oxidase* enzyme as both reducing and stabilizing agent and demonstrated their application through multimodal sensing strategy for selective detection of cysteine (Cys). Three different methods namely fluorescence turn OFF–ON strategy, naked eye detection and electrochemical methods are used for Cys detection by employing FL–Au NPs as a common probe. In case of fluorescence turn–OFF method a strong interaction between Au NPs and thiol results in quenching of fluorescence due to replacement of *glucose oxidase* by Cys at neutral pH. Second mode is based on fluorescence switch–ON strategy where initial fluorescence is significantly quenched by either excess acid or base and further addition of Cys results in appearance of rosy-red and green fluorescence respectively. Visual colour change and fluorescence emission arises due to etching of Au atoms on the surface by thiol leading to formation of Au nanoclusters. Finally, electrochemical sensing of Cys is also carried out using cyclic voltammetry in 0.1 M PBS solution. These findings provide a suitable platform for Cys detection over a wide range of pH and concentration levels and hence the sensitivity can also be tuned accordingly.

Recently biomolecules assisted preparation of gold nanoparticles (Au NPs) and nanoclusters (NCs) have received a great deal of attention in the field of nanoscience and technology due to their small size and bio-compatibility along with their intrinsic and exciting physical, chemical and optical properties. Hence, these NPs could be used in multifaceted applications. Upon drastically reducing the size of these NPs to clusters they exhibit remarkable fluorescence (FL) primarily due to the quantum confinement effect associated with sub-nanometre size of the particle^1^. Therefore, fluorescent NPs have been used in various fields such as detection of metal ions (Cu^2+^, Hg^2+^ and CH_3_Hg^+^) based on FL quenching or enhancement^2^,^3^,^4^, detection of H_2_O_2_^5^, trypsin^6^, melanine^7^, phosphate containing metabolics and *in vivo* bioimaging of cell markers etc^8^,^9^,^10^. The electronic and optical properties of these Au NPs mainly depend upon their size, especially in the nanometre size regime. In case of Au NPs possessing a relative core size in the range of 5–100 nm, the mean free path of light in this range exhibits a characteristic of surface plasmon resonance (SPR) feature attributed to the collective oscillation of conduction electrons upon interaction with light. Suppose the core size of Au NPs is below 2 nm, down to a few atoms, the band structure becomes discontinued and its broken down into discrete energy levels, displaying molecule-like behaviour and do not exhibit any SPR properties and hence emits fluorescence while exposing to UV light^11^,^12^. Similarly fluorescent property can also arise from the interaction of metal core of Au NPs with the specific surface ligand, in case where the size of these Au NPs is relatively larger than 2 nm. Then they exhibit SPR and fluorescence emission can be enhanced by the plasmonic resonance. Since the fluorophore is placed adjacent to metal NPs, their fluorescence intensity can be significantly altered due to increase in the radiative emission rate, which is caused by the increased local density of plasmonic states around NPs^13^,^14^.

By combining the striking feature of fluorescence properties along with other intrinsic chemical properties of these Au NPs it is possible to apply such nanomaterials in the fields of chemistry, materials and biology for a wide variety of applications. Large number of reports are available in literature discussing the origin of fluorescence for Au NPs and Au NCs^13^,^14^,^15^,^16^,^17^,^18^,^19^,^20^,^21^. Wu *et al*. reported the enhancement in fluorescence of Au NPs with the aid of specific surface ligands^13^. They have proposed the enhancement in fluorescence emission essentially originates from ligand to metal charge transfer (LMCT) and due to the presence of electron rich atoms (*eg*. N, O) or groups (−COOH, −NH_2_) of the surface ligand. Liü *et al*. demonstrated Au NPs functionalized with copolymer of 8–hydroxyquinoline (HQ) enhances the fluorescence because of the presence of abundant surface metal atoms^15^. Shang *et al*. prepared Au NPs stabilized with conjugated polymer (poly(9,9-bis(4¢-sulfnoatobutyl)fluorene-co-alt-1,4-phenylene)) that shows a weak fluorescence emission due to fluorescence resonance energy transfer between the fluorophore and Au NPs^16^. Similarly Muhammed *et al*. reported two distinct Au NCs such as Au_8_ and Au_25_ synthesized from mercaptosuccinic acid-protected Au NPs by etching process using glutathione at different pH values^17^. Briñas *et al*. demonstrated that the size of glutathione protected Au NPs could be controlled by varying pH of the solution between 5.5 and 8^18^. Schaeffer *et al*. examined the variation in size of Au NCs with respect to the concentration of polymer ratio used for protecting those clusters^19^. In addition to the size reduction resulting in the formation of metal NCs, the emission properties can also be varied from UV to near IR (NIR) region. Particularly this shift in emission wavelength could be exploited in many different research fields, such as single molecular detection and recognizing fluorescence of metal NCs by using optical sensing research^20^,^21^. Optically active noble metal nanomaterials such as Au and Ag are crucial for the construction of optical biosensors and these materials improve the properties in terms of cost, sensitivity, selectivity and miniaturization of devices etc.^22^,^23^,^24^. Recently glutathione protected Ag NPs have been shown as an optical sensor for the detection of cysteine (Cys) where the nanomaterial interacts with Cys and the optical response is measured by using colourimetric (with changing SPR of NPs) and fluorimetric methods^11^.

Cysteine (Cys; 2-amino-3-sulfhydrylpropanoic acid) is an important amino acid essentially consisting of a thiol (−SH) group apart from amine and carboxylic groups, which enables the bond formation between a metal surface and Cys containing proteins and peptide molecules. Also it is involved in many significant biological functions such as protein synthesis, detoxification and metabolism. Further it is widely used in many pharmaceutical products including some antibiotics, skin damage creams, neurotoxins, food additives, biomarkers for various medical conditions etc. Moreover, the quantitative determination of Cys is an indicative parameter of human and animal health conditions. Basically determination of Cys concentration level in physiological samples such as plasma, urine, and saliva can serve as a valuable biomarker in clinical applications. Concentration of Cys in these human body fluids typically varies between 5 μM and 30 μM. Deficiency of Cys and altered levels in human can cause genetic disorder such as homocystinuria, it is an inherited disorder in metabolism of the amino acid methionine, often involving cystathionine betasynthose. This defect leads to a multi-systemic disorder of the connective tissues, muscles in central nervous system (CNS) and cardio vascular system. Thus determination of Cys becomes important and critical. Therefore, the detection of Cys is very significant in day-to-day life and in the biological fields^25^,^26^,^27^,^28^.

Keeping this in mind, here we demonstrate the multimodal sensing of Cys by employing *glucose oxidase* (GOx) stabilized Au NPs that exhibits fluorescence (GOx–FL Au NPs) as a common probe. Simultaneously fluorescence emission behaviour of these Au NPs is tuned by altering pH of the solution. Figure 1 depicts the pictorial representation of the proposed strategy for multimodal sensing of Cys. Initially as-prepared GOx stabilized Au NPs (GOx–Au NPs) is in alkaline pH (pH = 10) and upon neutralization (pH = 7) using acid, GOx–Au NPs emits an intense yellowish green colour fluorescence. During incremental addition of Cys, fluorescence is quenched systematically due to the replacement reaction or phase exchange of GOx by the added Cys molecules as a result colour of the solution also changes from red to blue. Secondly, fluorescence of these NPs can also be drastically quenched with the aid of acid (tuning pH to acidic range of 5) and then subsequent addition of Cys leads to a colour change from red to violet. Surprisingly this mixed solution exhibits bright rosy-red colour fluorescence emission upon exposure to UV light. Similarly fluorescence of these Au NPs could also be deeply quenched by using a base (altering to alkaline pH of 10) and further addition of Cys results in a visual colour change from red to pale green that emits strong green colour fluorescence while irradiating with UV light. Finally, GOx–FL Au NPs solution is directly drop casted onto a glassy carbon (GC) electrode surface and electrochemical sensing of Cys is also carried out by using cyclic voltammetry (CV). Thus the proposed work identifies and demonstrates a suitable functional platform for tuning fluorescence using pH and simultaneously performs the multimodal sensing strategy using colourimetric, fluorimetric and electrochemical methods for the detection of Cys, a potential target analyte in biology, as represented in Fig. 1.

## Results and Discussion

### UV–Visible spectroscopic studies and TEM analysis

**Figure 2:**
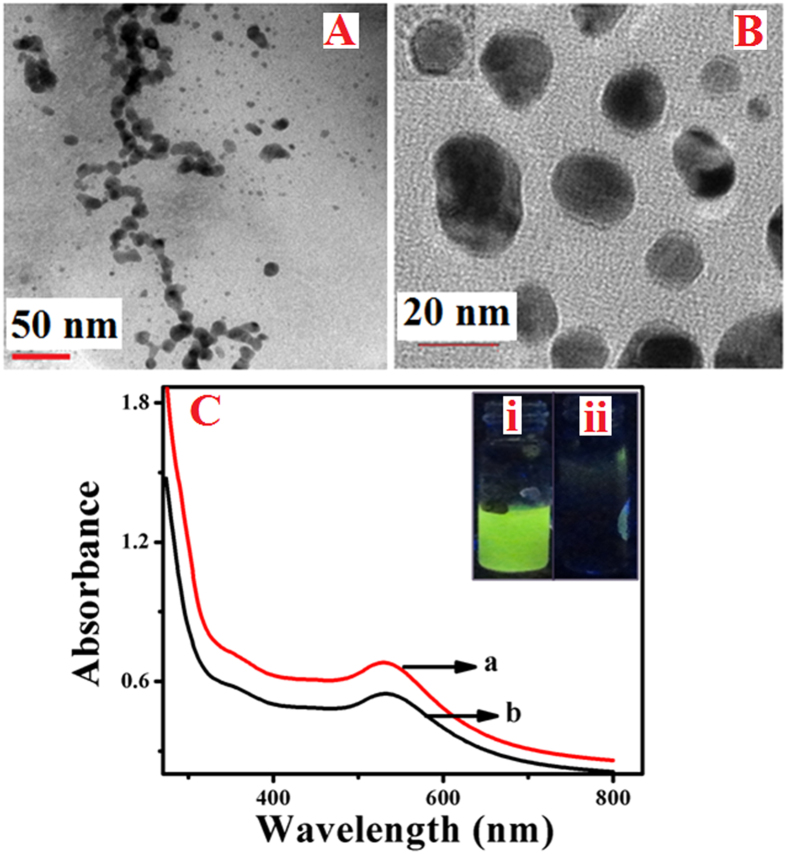
TEM images of as-prepared GOx–Au NPs (**A**) and GOx–FL Au NPs (**B**) obtained upon neutralization. (**C**) UV–Visible spectra of as-prepared GOx stabilized Au NPs (a) and GOx–FL Au NPs (b). Inset shows the photographs of GOx–FL Au NPs exhibiting a bright yellowish green fluorescence emission (i) and GOx–Au NPs (no fluorescence emission, ii) upon exposure to UV light.

In order to analyze the optical characteristics and functionalization associated with that of GOx enzyme stabilized Au NPs, UV–Visible spectroscopic studies are carried out and the corresponding spectra are shown in Fig. 2C. Initially, as-prepared GOx stabilized Au NPs are in alkaline medium (pH = 10) and after neutralization (pH = 7) these Au NPs exhibit a bright yellowish green fluorescence upon exposure to UV light. Their corresponding spectra are shown as “a” and “b” in Fig. 2C. Inset displays the photographs obtained for these solutions under UV illumination showing the appearance of yellowish green fluorescence behaviour upon neutralization. It can be visualized from the insets that as-prepared GOx stabilized Au NPs that are in alkaline pH, do not show any fluorescence (Inset–ii) and in contrary the same solution upon reducing the pH to neutral value display a bright yellowish green fluorescence (Inset–i). Further it can be noted from UV–Vis spectra that as-prepared GOx–Au NPs (a) and GOx–FL Au NPs (b) showed a prominent characteristic absorbance peak at 535 nm corresponding to the surface plasmon resonance (SPR) peak of Au NPs. Moreover, the characteristic absorption peak of oxidized form of flavin adenine di-nucleotide (FAD) present within the enzyme, GOx is also observed at 374 nm as a small hump (Supporting Information, Fig. S1).

Further the structure and morphology of the resultant NPs are analyzed using TEM studies. Figure 2A and B show the TEM images of GOx–Au NPs and GOx–FL Au NPs. It can be noticed that as-prepared GOx–Au NPs display an agglomerated structure in which the NPs are aligned in the polypeptide chain of protein network leading to the formation of “worm-like” morphology (Fig. 2A)^14^,^29^. In contrast, upon neutralizing the solution, these NPs are disintegrated to form smaller NPs (Fig. 2B) of size varying between 8 nm and 12 nm and the particle size distribution analysis suggests the average particle size is in the range of 9.6 ± 0.5 nm. Moreover, FTIR spectroscopy is also used for confirming the formation of GOx stabilized Au NPs. These results are in agreement with our earlier work wherein we described in detail the preparation of GOx stabilized Au NPs and demonstrated their application for dual mode sensing of glucose^14^. Interestingly upon neutralization of the resultant Au NPs, an intense yellowish green fluorescence is observed and the origin of fluorescence for GOx–FL Au NPs is explained based on the enhancement of fluorophore present within the enzyme with the aid of these Au NPs as reported in our previous work^14^. Basically in this case fluorescence originates from flavin adenine dinucleotide (FAD) and its oxidized form, FADH_2_ present within the enzyme. Interestingly this particular fluorescence behaviour is significantly enhanced through the interaction with Au NPs. Since the size of these Au NPs are relatively larger than 2 nm as observed from Fig. 2B, they do not exhibit fluorescence by themselves like Au NCs^14^. Instead these Au NPs display SPR peak and due to the interaction of surface plasmon with FAD fluorophore of the GOx enzyme the enhanced fluorescence is observed. This is due to the result of increased radiative emission rate caused by the increased local density of photonic states around the NPs that are placed adjacent to the fluorophore of the enzyme^14^. It has been reported that the fluorescence of FAD remains unaltered within the pH range of 5 to 9 and further increase in pH results in drastic decrease of fluorescence^30^. Basically FAD exists in the form of two conformers namely the stack conformer where stacking of adenine moiety and isoalloxazine ring occurs and the other is ‘unstack’ conformer, in which lifetime and fluorescence is unaffected. Mainly π−π stacking interaction between aromatic rings and intramolecular hydrogen bonds along the phosphate sugar backbone gives the stabilization for this stack conformer. Many spectroscopic techniques have been used to explore this ‘stack–unstack’ behaviour of FAD. Moreover, the pH dependence of fluorescence behaviour of FAD has also been studied^31^,^32^,^33^. It is found that the fluorescence remains unaltered for pH ranging between 5 and 9. Further increase in pH shows a weak fluorescence for FAD. These reports agree well with our observations presented in the current work. Subsequently, in this work, we demonstrate a multimodal sensing of Cys using a similar GOx–FL Au NPs by tuning the fluorescence behaviour with pH thereby, detection of Cys is carried out under acidic, neutral and alkaline pH values.

### Detection of Cys under neutral condition (pH = 7) using GOx–FL Au NPs

**Figure 3:**
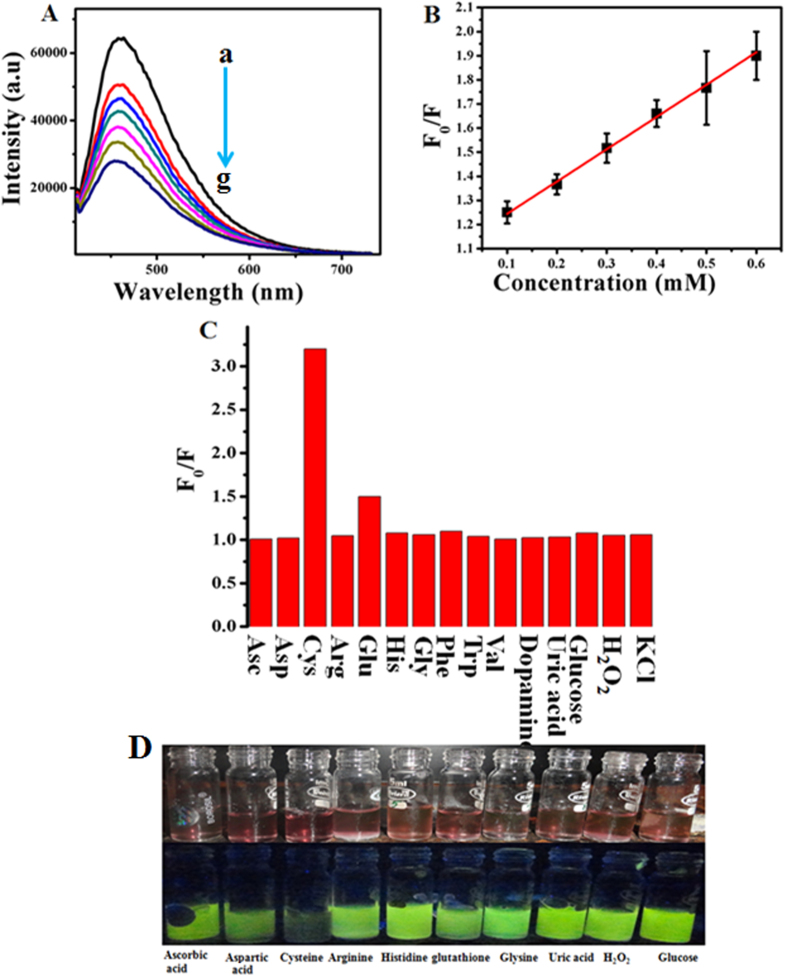
(**A**) PL emission spectra of GOx–FL Au NPs recorded in presence of various concentrations of Cys namely (a) 0 mM, (b) 0.1 mM, (c) 0.2 mM, (d) 0.3 mM, (e) 0.4 mM, (f) 0.5 mM and (g) 0.6 mM respectively. Arrow indicates the direction of increasing concentration of Cys. (**B**) A plot of relative change in fluorescence intensity (F_0_/F) of GOx–FL Au NPs vs. concentration of Cys. Error bars represent the standard deviation of three independent measurements. (**C**) Comparison of fluorescence quenching efficiency of GOx–FL Au NPs in presence of Cys and various other potential interferences. (**D**) Photographs of GOx–FL Au NPs solution with the addition of fixed concentration (0.1 mM) of different amino acids and other potentially interfering bio-molecules before and after illumination under UV (365 nm) lamp.

Interestingly as-prepared colloidal red coloured solution of GOx–Au NPs and GOx–FL Au NPs obtained upon neutralization possess a remarkable stability in aqueous solution under ambient conditions. These NPs solutions are found to be stable for more than 12 months. The first mode of Cys detection is carried out using fluorescence turn OFF strategy under neutral pH. GOx–FL Au NPs exhibits a bright yellowish green fluorescence emission at neutral pH and upon addition of Cys, initially fluorescence is gradually decreased and it is eventually quenched at very high concentrations. Figure 3A shows the fluorescence emission spectra recorded using GOx–FL Au NPs for addition of various concentrations of Cys and for comparison a similar spectrum recorded in absence of Cys is also provided. It can be noticed that GOx–FL Au NPs exhibit a strong emission peak at 445 nm upon excitation at a wavelength of 360 nm. Further addition of Cys leads to a systematic decrease in fluorescence intensity and at higher concentrations the complete fluorescence quenching is observed. Moreover, UV–Visible absorption spectra are also recorded using this colloidal red coloured solution of GOx–FL Au NPs for the addition of different concentrations of Cys (Supporting Information, Fig. S2). The absorption peak observed at 535 nm is greatly altered in the presence of Cys along with the decrease in absorbance value is noted and a new peak is appeared at 660 nm (Fig. S2). These observations clearly support the evidence for systematic quenching of fluorescence with respect to increasing addition of Cys. The optical properties of fluorescence quenching and absorption bleaching of GOx–FL Au NPs arise mainly from the interaction between Cys and Au NPs. This is due to the replacement of GOx present on the surface of these Au NPs by Cys and forms a strong Au–S interaction leading to aggregation of Au NPs resulting in the formation of a larger sized Au NPs^29^,^34^. Hence it suppressed the plasmonic enhancement caused by Au NPs resulting in the significant quenching of fluorescence. Aggregation of Au NPs is further confirmed by HRTEM analysis and the images clearly show the formation of aggregated Au NPs upon Cys addition to form a chain-like structure (Supporting Information, Fig. S3).

Figure 3B shows a plot of relative change in fluorescence intensity (F_0_/F) with respect to concentration of Cys added. Here F_0_ represents the fluorescence of GOx–FL Au NPs before adding Cys and F designates the fluorescence intensity measured for each and every addition of Cys. It can be noticed that the intensity ratio is linearly increased with increasing concentration of Cys. It exhibits a perfect linear relation for a wide concentration of Cys ranging from 0.1 mM to 0.6 mM with a linear regression coefficient of R^2^ = 0.9919. Further fluorescence quenching with respect to increasing amount of Cys is analyzed using Stern–Volmer equation given below^35^.




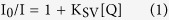




where I_0_ and I are the fluorescence intensity values measured at 445 nm in the absence and presence of Cys, K_SV_ is the Stern–Volmer fluorescence quenching constant and [Q] is the concentration of quencher (Cys) respectively. K_SV_ is calculated from the linear regression plot (Fig. 3B) and the value is found to be 0.4 mM^−1^ and the lowest detection limit is determined to be 50 μM. In addition, reproducibility is investigated by recording the emission spectra for three consecutive measurements and the obtained response suggests a good reproducibility for the system.

Similarly, selectivity of the proposed Cys detection is studied by using various other amino acids namely glycine (Gly), arginine (Arg), phenylalanine (Phe), tryptophan (Trp) and histidine (His) along with the other potentially interfering biological molecules such as glucose, hydrogen peroxide (H_2_O_2_), dopamine, ascorbic acid and uric acid (Fig. 3C) by recording their respective fluorescence emission spectra. Figure 3D shows the photographs of GOx–FL Au NPs solution for 0.1 mM addition of all the above compounds before and after illumination with UV light (365 nm). It is clearly evident from Fig. 3C that these molecules showed a very negligible interference for Cys detection. Moreover these interference substances did not show any visible colour change even at higher concentration levels (Fig. 3D). But, in the case of glutathione (GSH) which also contains a thiol group, a little fluorescence change is observed and the colour also varied after a longer time. Interestingly at higher concentration levels of GSH the observed change in fluorescence intensity is very minimum due to steric effect which restricted the adsorption of GSH on the surface of Au NPs^36^. Nevertheless the bright yellowish green fluorescence emission is retained in all the cases except Cys, suggesting a potential fluorescence turn–OFF sensing method for Cys detection. These results clearly indicate a potential utility of GOx–FL Au NPs for quantitative determination of biologically important molecule like Cys at neutral pH.

### Fluorescence turn OFF–ON based Cys detection using GOx–FL Au NPs under acidic (pH = 5) and basic (pH = 10) conditions

**Figure 4:**
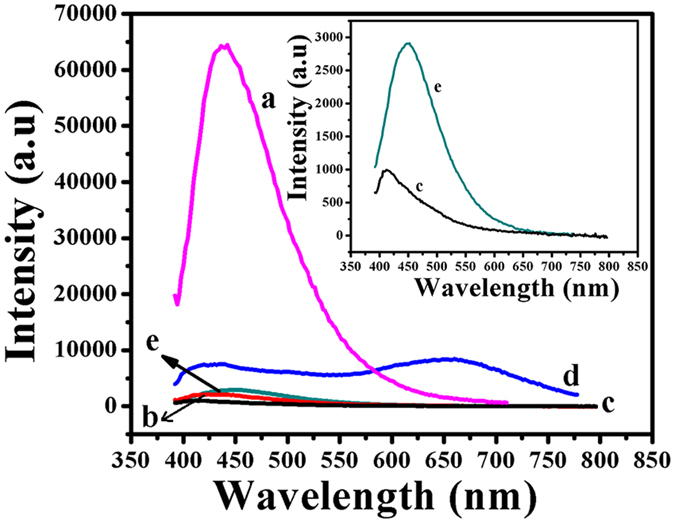
Fluorescence emission spectra recorded using GOx–FL Au NPs under neutral (pH = 7) (**a**), acidic (pH = 5) (**b**) and basic (pH = 10) (**c**) conditions before the addition of Cys. Similarly fluorescence spectra recorded for GOx–FL Au NPs under acidic (**d**) and basic (**e**) conditions after the addition of a fixed concentration of Cys are also provided. Inset shows the emission spectra recorded under basic condition for GOx–FL Au NPs before (**c**) and after the addition (**e**) of Cys.

From our studies we find that pH is one of the main factors which influence the fluorescence behaviour of enzyme stabilized Au NPs. On changing the pH value from neutral where these NPs exhibits a bright fluorescence emission to either acidic or basic condition, these NPs show a very weak fluorescence emission upon exposure to UV light. In general UV–Visible and fluorescence spectroscopic techniques are one of the most important spectroscopic techniques to evaluate the structural and optical properties of nanomaterials. UV–Visible absorption spectra recorded for GOx–FL Au NPs under acidic (pH = 5) and basic (pH = 10) conditions (Supporting Information, Fig. S4A) reveal that these NPs retain SPR absorption peak of Au NPs along with the characteristic features of the enzyme, GOx. In case of acidic condition this peak is shifted to 530 nm and in basic medium it appeared at 525 nm. Interestingly under these conditions, fluorescence property associated with the NPs is significantly suppressed and it shows only a feeble fluorescence. Subsequently upon addition of Cys, these NPs surprisingly display a strong fluorescence emission under illumination with UV light. Under acidic condition, addition of Cys results in rosy-red fluorescence and at basic pH, it emits an intense green colour. Further UV–Visible and fluorescence spectroscopic studies are carried out for these Au NPs in presence of Cys under both the acidic and basic conditions. UV–Vis spectra showed that the characteristic SPR peak is completely disappeared in both the cases and two new peaks are formed at 336 nm and 407 nm for acidic pH and similarly the peaks appeared at 350 nm and 398 nm for basic medium respectively (Supporting Information, Fig. S4B). These new peaks appeared in the lower wavelength (blue) region suggesting a possible reduction in the size of these NPs leading to the formation of Au NCs. This is also supported by the disappearance of prominent SPR peak in both the cases. Figure 4 shows the fluorescence emission spectra recorded at a fixed excitation wavelength of 360 nm for all the cases discussed above. It can be noted that at neutral pH, GOx–FL Au NPs display a strong emission at 445 nm (yellowish green fluorescence) (Fig. 4a) and upon changing pH to acidic (Fig. 4b) and basic (Fig. 4c) conditions, the emission spectra showed no peaks and the corresponding fluorescence is significantly decreased. Subsequently upon adding Cys, these NPs exhibit fluorescence again under both the acidic and basic conditions. On adding Cys, GOx–FL Au NPs show a rosy-red colour fluorescence having a maximum emission at a wavelength of 650 nm under acidic condition (Fig. 4d). Similarly under basic condition, these NPs show a green colour possessing a maximum emission at 445 nm on adding Cys (Fig. 4e). Compared to the decreased fluorescence system, addition of Cys in acidic condition results in ~4–5 fold increase in fluorescence intensity whereas in basic condition only about 3 fold enhancement in the corresponding fluorescence intensity is observed. Further excitation wavelength dependency on this fluorescence emission behaviour is investigated for both acidic and basic conditions upon adding a fixed concentration of Cys (Supporting Information, Figs S5 and S6). Under acidic (pH = 5) condition, excitation wavelength is varied between 330 nm and 400 nm and fluorescence emission spectra are recorded. A maximum rosy-red fluorescence emission is observed at 650 nm for an excitation wavelength of 360 nm (Fig. S5) and these GOx–FL Au NPs show excitation wavelength dependent emission behaviour. Similarly for basic (pH = 10) condition, a maximum green fluorescence emission is observed at 445 nm for an excitation wavelength of 360 nm (Fig. S6). This fluorescence tuning property associated with GOx–FL Au NPs upon altering pH is explored further for the detection of Cys using fluorescence turn OFF–ON strategy.

**Figure 5:**
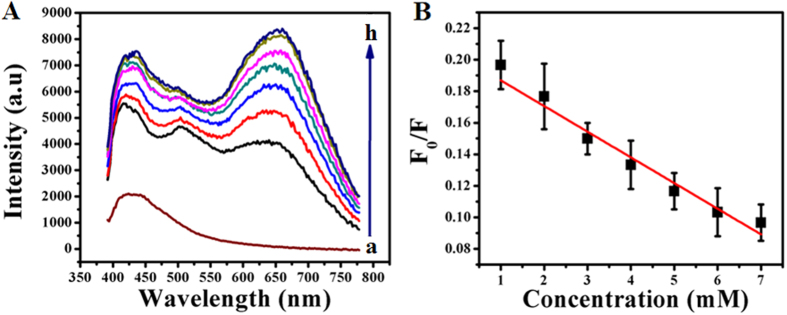
(**A**) Fluorescence emission spectra of GOx–FL Au NPs recorded at an excitation wavelength of 360 nm under acidic (pH = 5) condition for the addition of different concentrations of Cys namely (a) 0 mM, (b) 1 mM, (c) 2 mM, (d) 3 mM, (e) 4 mM, (f) 5 mM, (g) 6 mM and (h) 7 mM respectively. Arrow indicates the direction of increasing concentration of Cys addition. (**B**) A plot of relative change in fluorescence intensity (F_0_/F) of GOx–FL Au NPs as a function of added Cys concentration. Error bars indicate the standard deviation calculated from three independent measurements.

Figure 5A shows the fluorescence emission spectra recorded using GOx–FL Au NPs under acidic (pH = 5) condition along with the addition of different concentrations of Cys at a fixed excitation wavelength of 360 nm. For comparison a similar spectrum recorded in the absence of Cys is also shown (a) and it displayed a very low fluorescence emission upon exposure to UV light indicating the significant quenching of fluorescence on adding acid. Concentration of Cys is varied between 1 mM and 7 mM. It can be seen that on adding Cys, a new fluorescence emission peak is observed at 650 nm and it systematically increases with increasing concentration of Cys. Upon irradiation with UV light this solution emits a rosy-red fluorescence (Fig. S5). The relative change in fluorescence intensity (F_0_/F, where F_0_ represents the fluorescence of GOx–FL Au NPs before adding Cys and F designates the fluorescence intensity measured for each and every addition of Cys in acidic pH value) with respect to concentration of Cys is noted down and the corresponding plot is shown in Fig. 5B. Notably, this ratio linearly decreases with incremental addition of Cys. From this plot a lowest detection limit of 89 μM for Cys sensing is calculated. Reproducibility of this fluorescence turn ON sensor is investigated by repeating the experiment thrice and the error bars in Fig. 5B represent the standard deviation value obtained from three consecutive and independent measurements.

**Figure 6:**
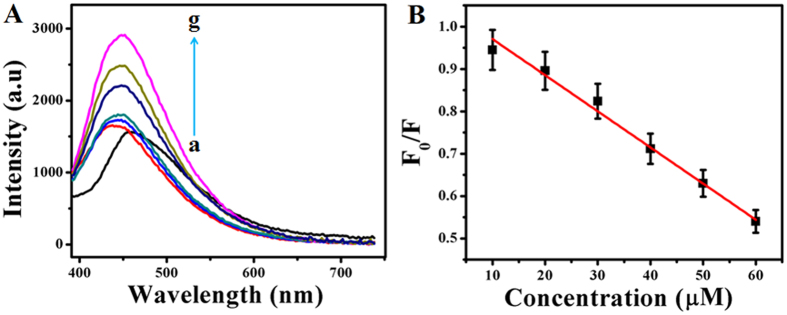
(**A**) Fluorescence emission spectra of GOx–FL Au NPs recorded at an excitation wavelength of 360 nm under basic (pH = 10) condition for the addition of different concentrations of Cys namely (a) 0 μM, (b) 10 μM, (c) 20 μM, (d) 30 μM, (e) 40 μM, (f) 50 μM and (g) 60 μM respectively. Arrow indicates the direction of increasing concentration of Cys addition. (**B**) A plot of relative change in fluorescence intensity (F_0_/F) of GOx–FL Au NPs as a function of added Cys concentration at basic pH. Error bars indicate the standard deviation value obtained from three independent measurements.

Similarly the fluorescence associated with GOx–FL Au NPs is also quenched by using NaOH solution by adjusting the pH value to 10 (alkaline) and interestingly upon adding Cys, this solution emits a bright green colour fluorescence when irradiating under UV light (Fig. S6). Figure 6A displays the fluorescence emission spectra recorded using GOx–FL Au NPs under basic (pH = 10) condition along with the addition of various concentrations of Cys at a fixed excitation wavelength of 360 nm. For comparison a similar spectrum is also recorded in the absence of Cys in basic condition and it displayed a negligible fluorescence upon exposure to UV light indicating the large fluorescence quenching on adding a base. Concentration of Cys is varied between 10 μM and 60 μM. It can be seen that on adding Cys, a new fluorescence emission peak is observed at 445 nm which is shifted to blue region and it systematically increased with increasing concentration of Cys. Figure 6B shows the relative change in fluorescence intensity (F_0_/F, where F_0_ represents the fluorescence of GOx–FL Au NPs before adding Cys and F denotes the fluorescence intensity measured for each and every addition of Cys in basic condition) with respect to increasing concentration of Cys. It can be observed that the ratio decreased linearly with incremental addition of Cys and a lowest detection limit is calculated to be 6.8 μM. Furthermore reproducibility of this fluorescence turn–ON Cys detection in basic medium is analyzed by repeating the experiment thrice and the error bars in Fig. 6B designate the standard deviation value calculated from three consecutive and independent measurements.

### Naked eye detection of Cys using GOx–FL Au NPs

Apart from fluorescence quenching and fluorescence turn OFF–ON methods, naked eye detection of Cys is also proposed in this work. Particularly the advantage of this method involves visualizing the colour change with naked eye under a wide pH range covering acidic, neutral and alkaline conditions. As mentioned earlier, as prepared GOx–Au NPs is in alkaline medium and upon neutralization these NPs emit a strong yellowish green fluorescence. On adding Cys under neutral condition, this fluorescence behaviour is systematically quenched and concomitantly the colour of these NPs solution changed from red to blue (Supporting Information, Fig. S2) due to the occurrence of phase exchange reaction leading to aggregation of Au NPs. UV–Visible absorption studies clearly show that GOx–FL Au NPs display a prominent SPR peak at 535 nm in neutral pH and upon incremental addition of Cys, this particular absorption peak at 535 nm decreases and a new peak is appeared at 660 nm whose absorption value is systematically increased. Consequently the colour of the solution changed from red to blue on varying the concentration of Cys from 10 μM to 80 μM in this case (Fig. S2). Quantitative measurement of absorption ratio of these two peaks (A_660_/A_535_) shows a steady increase in the beginning and attains saturation at higher concentration of Cys (Fig. S2). Similarly under acidic (pH = 5) condition, on adding Cys the colour of the solution changes from red to violet and for basic medium, the colour changes from red to pale green. Concentration of Cys is varied between μM and mM. UV–Visible absorption spectra of the resulting mixture showed increase in absorption value with increase in the concentration of Cys (Supporting Information, Fig. S7). Thus the proposed method covers a wide range of concentration and a wide range of pH value for Cys detection using naked eye colour change strategy.

### Mechanism behind Cys sensing using GOx–FL Au NPs

In this work, we demonstrated multimodal sensing methods for Cys detection using GOx–FL Au NPs by employing colourimetric, fluorimetric and electrochemical methods. Interestingly the fluorescence emission behaviour of these NPs could well be tuned by altering pH of the medium along with the addition of Cys. The mechanism of fluorescence switching in presence of Cys under both the acidic and basic conditions is still unclear and possible explanation could be provided based on our studies and it is attributed mainly to the formation of Au NCs. According to literature reports, there are two possible routes for the formation NCs under acidic and basic conditions^17^,^18^. In the first route, removal of gold atoms from the surface of Au NPs in presence of excess of Cys molecules resulting in the formation of gold (I) Cys thiolate complex is proposed. Due to the strong aurophilic interaction of this complex, formation of either a dimer or oligomer chains is possible through hybridization of empty 6s/6p and filled 5d orbitals resulting in Au (I)–Au (I) interaction within the layers leading to formation of lower sized particles of Cys protected Au NCs^17^,^18^. In the second possible route, etching of gold atoms from the surface of Au NPs occurs with the aid of thiol group present within Cys molecule leading to the reduction in size causes the formation of Au NCs. These two possible routes are also applicable in both acidic and basic media^17^,^18^. Similarly, the size of Au NPs can also be controlled by the use of polymeric Au (I) thiolate precursor with varying pH, generally lower pH values favour larger and denser polymeric precursors resulting in the formation of larger sized Au clusters and the higher pH values favour smaller and less dense precursors leading to the formation of smaller Au clusters. In both cases Au (I) thiolate intermediate is formed during the etching^18^. The same mechanism is applicable in the present system, where under the acidic condition in presence of Cys, GOx–FL Au NPs aggregate among themselves resulting in the formation of larger sized clusters and hence the colour of GOx–FL Au NPs changes from red to light violet. Consequently, the fluorescence of enzyme stabilized Au NPs is red shifted. On contrary, under the basic condition in presence of Cys, the colour of GOx–FL Au NPs changes from red to green and the corresponding fluorescence emission spectra is blue shifted. Hence based on our observations and from the results of spectroscopic and microscopic studies, a possible mechanism is proposed in which GOx–FL Au NPs disintegrate to form Au NCs. This is further confirmed by TEM, mass spectroscopic and UV–Vis spectroscopic analyses.

TEM images and mass spectroscopic data are recorded for these NPs after the addition of Cys under both acidic and basic conditions respectively (Supporting Information, Fig. S8 to Fig. S10). Compared to the size of these Au NPs determined at neutral pH (~9.6 nm), the average size of these Au NPs after the addition of Cys in acidic and basic conditions is hugely reduced and it is determined to be 2–2.5 nm ± 0.2 nm. Moreover, mass spectroscopic analysis clearly reveal the formation of smaller Au NCs of size between Au_3_ and Au_8_ functionalized with Cys and is attributed mainly to the surface etching of Au atoms by thiol group^34^. These results are also in good agreement with UV–Visible spectroscopic observations where the SPR peak corresponding to Au NPs is completely disappeared after the addition of Cys (Fig. S4B) since the work-function of NCs did not match with that of the normal metallic state. Moreover, the size of Au NCs matches with Fermi wavelength of free electrons leading to the formation of quantized energy states and hence the energy levels of these NCs equal the molecule like behaviour^20^,^35^. Thus it enables a multimodal sensing strategy for the detection of Cys over a wide concentration range including the biologically relevant concentration range under neutral, acidic and basic conditions.

### Electrochemical detection of Cys using GOx–FL Au NPs modified GC electrode

**Figure 7:**
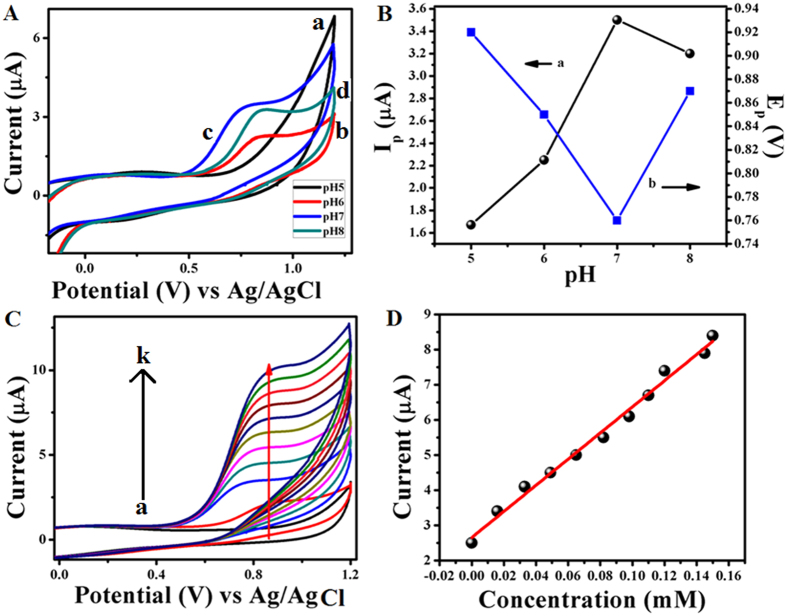
(**A**) CVs corresponding to GOx–FL Au NPs modified GC electrode in different pH solutions (0.1 M PBS) namely pH = 5 (a), 6 (b), 7 (c) and 8 (d) respectively consisting of 1.0 mM Cys at a fixed scan rate of 50 mV/s. (**B**) Plots of I_p_ (a) and E_p_ (b) corresponding to electrochemical oxidation of Cys vs. pH. Data points are obtained from Figure A. (**C**) CVs of GOx–FL Au NPs modified GC electrode in 0.1 M PBS solution at a potential scan rate of 50 mV/s for addition of various concentrations of Cys viz., (b) 0.016 mM, (c) 0.033 mM, (d) 0.049 mM, (e) 0.065 mM, (f) 0.082 mM, (g) 0.098 mM, (h) 0.11 mM, (i) 0.12 mM, (j) 0.145 mM and (k) 0.15 mM respectively. For comparison similar CV recorded in the absence of Cys (a) is also shown. (**D**) A plot of variation in current with respect to added Cys concentrations. Data points are obtained from Figure C.

Further electrochemical detection of Cys is also carried out by modifying GC electrode with GOx–FL Au NPs and the sensing events are investigated by monitoring the oxidation of Cys using cyclic voltammetry. These experiments are carried out in an aqueous solution consisting of 0.1 M PBS buffer within the potential ranging from −0.1 V to 1.2 V at a fixed scan rate of 50 mV/s. Typical cyclic voltammograms obtained are shown in Fig. 7. Initially pH of the electrolyte solution is optimized for Cys detection. Figure 7A shows the voltammograms recorded using GOx–FL Au NPs modified GC electrode towards Cys detection under various pH values namely 5, 6, 7 and 8. It can be noticed that the modified electrodes show a clear peak in CV corresponding to Cys oxidation and this particular oxidation peak appears at relatively a lower oxidation potential with higher peak current at neutral pH when compared to all the other pH values. The change in peak current (I_p_) and peak potential (E_p_) values corresponding to Cys oxidation with respect to change in pH values are plotted in Fig. 7B. It can be clearly seen that the peak current is maximum (3.5 μA) and peak potential is minimum (0.75 V) at neutral pH whereas the peak current decreases in other pH values and peak potential (E_p_) values shift to higher oxidation potential (more positive) values for other pH ranges. Taking into consideration the higher peak current response, lower oxidation potential under the normal physiological conditions, pH = 7 is chosen for investigating the electrochemical sensing of Cys and subsequently the experiments are carried out under these conditions. Basically in our work the oxidation of cysteine occurs at a higher positive potential of 0.75 V vs. Ag/AgCl. Compared to many other reported values this particular oxidation potential is higher. But it has some advantages like at this potential selectivity could be achieved since many small biologically relevant small molecules like dopamine, ascorbic acid, uric acid, hydrogen peroxide and glucose etc. oxidize at very lower potential and hence interference can be avoided. Secondly, this particular modified electrode shows a clear oxidation peak for Cys at higher potential which otherwise would be masked by water oxidation reaction. This peculiar characteristic might be useful for the detection of bio-analytes having a higher oxidation potential. Similar observations are reported in literature earlier^37^,^38^. Figure 7C represents the CVs of GOx–FL Au NPs modified GC electrode in 0.1 M PBS aqueous solution (pH = 7) at a fixed scan rate of 50 mV/s for addition of various concentrations of Cys. A concentration range of 16 μM to 0.15 mM is used for the study. For comparison a similar CV recorded in the absence of Cys is also shown where no oxidation peak is formed. It is evident from the CVs that the oxidation current corresponding to Cys oxidation increases systematically with incremental addition of Cys suggesting a good catalytic activity of the modified electrode towards Cys detection. Furthermore a calibration curve is plotted by measuring the variation in oxidation current with respect to added Cys concentrations and the corresponding plot is shown in Fig. 7D. This particular plot shows a good linear relationship between the measured current and Cys concentration from which a linear concentration range of 0.016 mM to 0.150 mM and a sensitivity value of 0.0372 μA/mM are determined along with a lowest detection limit of 1.55 μM. These values suggest a possible application of GOx–FL Au NPs as an electrochemical probe for the detection of Cys. In order to prove that GOx–FL Au NPs aid in electrochemical oxidation of Cys several control experiments are carried out. Initially bare GC electrode without surface modification does not show any oxidation peak corresponding to Cys in 0.1 M PBS solution, suggesting its inability to oxidize Cys within the potential range used for the study. Further different kinds of bulk gold electrodes namely polycrystalline and single crystalline gold are investigated for Cys oxidation and the corresponding CVs are recorded (Supporting Information, Fig. S11). It is noticed that these electrodes display characteristic features corresponding to gold oxide formation and reduction with a small hump corresponding to Cys oxidation at higher potential values. Among the two only single crystal gold electrode shows a distinguishable peak for electrochemical oxidation of Cys. In contrast, GOx–FL Au NPs modified GC electrode shows a clear peak at lower potential for Cys oxidation. Moreover, the current value is higher in this case, suggesting an enhanced activity for Cys oxidation. Furthermore, selectivity study is also carried out using several interfering ions namely glucose, hydrogen peroxide, ascorbic acid, uric acid, dopamine, glutathione etc. by following chronoamperometric study, where the change in oxidation current of these ions are monitored with respect to added concentration at a fixed potential (Supporting Information, Fig. S12). Since the oxidation potential values of these ions are distinctly different from the Cys oxidation potential, there is no interference due to these ions for Cys detection. These experiments clearly prove that these Au NPs help in selective electrochemical oxidation of Cys.

Interestingly, in this work we demonstrated a multimodal sensing strategy for the detection of Cys using GOx–FL Au NPs as a common probe. Typical sensor characteristics associated with the proposed sensing methods are compared with the other reported methods and the comparison is provided in Table 1^ ^16,39,40,41,42,43,44,45,46,47,48,49,50,51,52,53,54,55. This comparison suggests the proposed material exhibits a comparable and in some cases even better detection limit and sensitivity values than the other reported methods and materials. Moreover the limit of detection, linear concentration range and sensitivity values reported in the present work for various methods are in good agreement with the necessary biological range of Cys detection.

## Conclusions

In the present work, *glucose oxidase* enzyme stabilized fluorescent Au NPs have been successfully prepared and applied for the detection of Cys through a multimodal sensing pathway by using colourimetric, fluorimetric (turn–OFF and turn–ON modes) and electrochemical methods. A new fluorescent probe is developed towards the sensing of Cys under various pH values (acidic, neutral and basic conditions). Interestingly fluorescence emission colour of the resultant NPs could be tuned by adjusting the pH values and with the aid of Cys addition resulting in an intense fluorescence emission due to the formation of nanoclusters arising from the strong etching of Au atoms by thiol moiety present in Cys. Sensitive and selective detection of Cys using GOx–FL Au NPs is achieved because of the unique and strong Au–S interaction. These Au NPs possess a superior selectivity for Cys over the other amino acids and some common potentially interfering biological molecules. These results clearly reveal that GOx–FL Au NPs is a potential candidate for Cys detection using fluorescence turn OFF–ON switching mode depending upon the pH and naked eye sensing using colourimetric method. Moreover, GOx–FL Au NPs modified GC electrode shows an excellent electrocatalytic activity towards Cys at lower oxidation potentials in neutral pH when compared to bare GC electrode. Currently in our lab further work is underway to explore other applications in the fields of enzymatic fuel cells and some biologically important crucial target analytes.

## Methods

### Chemicals

Enzyme, *Glucose Oxidase* (GOx) extracted from Aspergillus Niger was purchased from Sigma Aldrich, Bangalore, India. AuCl_3_.HC1.4H_2_O was purchased from Sigma Aldrich. Glucose and cysteine (Cys) were obtained from HiMedia. Similarly KH_2_PO_4_ & K_2_HPO_4_ (HiMedia), NaOH (Merck) and HCl (Merck) were also procured. All these chemicals were of analytical grade (AR) and used as received without any further purification. Millipore water having a resistivity of 18.2 MΩ cm was obtained from a quartz distillation unit and used for the preparation of all the aqueous solutions employed in this work.

### Preparation of GOx stabilized fluorescent gold nanoparticles (GOx–FL Au NPs)

Prior to the preparation, all the glassware were cleaned with aqua regia; rinsed with millipore water and then dried in air. (*Caution:* While handling aqua regia special care must be taken for precautions, because of its highly toxic and corrosive nature). In a typical experiment, about 0.01 M NaOH was dissolved in 5 ml of millipore water. Then the resulting solution was stirred in an ice bath and GOx (~1 mg/ml) enzyme was mixed followed by the addition of 1 mM AuCl_3_ aqueous solution. During this process, colour of the solution changed from yellow to red indicating the formation of GOx stabilized Au NPs (GOx–Au NPs)^14^. Interestingly pH of the resultant solution was identified to be alkaline (pH = 10) and upon neutralization by adding a few drops of 0.1 M HCl to bring down the pH to 7, formation of tiny Au NPs was observed and interestingly these NPs showed an intense yellowish green colour fluorescence upon exposure to UV light and designated as GOx–FL Au NPs. Structure and morphology of the resultant NPs along with their optical properties were analyzed using microscopic and spectroscopic characterization techniques.

### Fluorescence turn OFF–ON and naked eye detection of Cys using GOx–FL Au NPs

Aqueous solutions of Cys with different concentrations were freshly prepared just before use. In case of fluorescence quenching studies, different concentrations of Cys solution were introduced separately into 1 mL of GOx–FL Au NPs solution. After the incubation of about 30 minutes, fluorescence emission spectra were recorded and simultaneously the colour changes were also monitored using UV–Visible spectroscopy. Initially fluorescence of these NPs was significantly altered through quenching either by using 0.1 M HCl to tune the pH into an acidic range (pH = 5) and likewise by using 0.1 M NaOH solution to obtain a basic pH value of 10. Then the various concentrations of Cys were added into the resultant solution and incubated at 37 °C for about 30 minutes. Finally fluorescence of the mixed resultant solution was recorded using a multiplate reader for each and every concentration of the added analyte.

### Preparation of electrochemical sensor electrode

Further, electrochemical detection of Cys was also performed using CV by employing GOx–FL Au NPs as a sensing probe. Before modification, GC electrode was polished with alumina powder of progressively smaller size and ultrasonicated in millipore water for about 15 minutes. Then the pre-cleaned GC electrode was analyzed by using CV in 1 mM of K_4_[Fe(CN)_6_] (a redox probe) and 0.1 M NaCl (supporting electrolyte) aqueous solution for background checking and for analyzing the surface cleanliness of the electrode. Then the electrode was washed with plenty of water and dried in air. Further the polished GC electrode was modified with GOx–FL Au NPs by drop casting them onto GC surface and dried overnight at room temperature. The resulting modified electrode was directly dipped into the Nafion (0.1%) solution for 1–2 minutes and then dried under N_2_ gas. Finally, GOx–FL Au NPs modified GC electrode was used for electrochemical sensing of Cys by employing CV studies in 0.1 M phosphate buffer solution (PBS) to monitor the binding events.

### Instrumentation

Electrochemical experiments were carried out using Biologic equipment (Model No. SP–240) procured from France. The corresponding experiments and their analyses were carried out using EC lab software provided by them. Absorbance and fluorescence spectra were recorded by employing UV–Vis Perkin Elmer Lamda 650 with Infinite M200MPC model. TEM and HRTEM images were obtained using TECNAI G^2^ 20 FEI model operated at 200 kW. TEM samples were prepared by drop casting method on a copper grid. FTIR spectra were recorded using Bruker Optic GmbH TENSUR 27 model operated with a 162 software Opus version 6.5 m.

## Additional Information

**How to cite this article**: Muthurasu, A. and Ganesh, V. Multimodal Sensing Strategy Using pH Dependent Fluorescence Switchable System. *Sci. Rep.* **6**, 39452; doi: 10.1038/srep39452 (2016).

**Publisher's note:** Springer Nature remains neutral with regard to jurisdictional claims in published maps and institutional affiliations.

## Supplementary Material

Supplementary Information

## Figures and Tables

**Figure 1:**
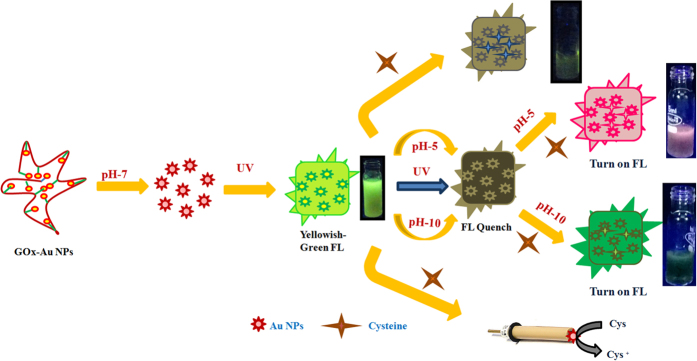
Pictorial representation of tuning the optical and fluorescence properties of enzyme stabilized Au NPs using pH and utilization of this strategy for multimodal detection of Cys.

**Table 1:** Comparison of limit of detection (LOD) and sensitivity values obtained in the present work using GOx–FL Au NPs as a probe for Cys detection with that of the other reported materials and methods.

Colorimetric method	LOD	Sensitivity	References
Citrate Au NPs	10 nM	—	39
Ag NPRs	25 nM	—	40
Au–CEQC	40 nM	—	41
CMC–Au NPs	80 μM	—	42
FSN–capped Au NPs	66.7 μM	—	43
Au NPs	0.01 ppm	—	44
GOx–FL Au NPs	37 μM	—	Present work
**Fluorescence turn−OFF method**	**LOD**	**Sensitivity**	**References**
SG–Hg^2+^− MSD	0.0034 μM	—	45
GSH–Ag NCs	10 nM	—	46
PMAA–Ag clusters	20 nM	—	36
AIE materials	0.5 μM	—	47
GOx–FL Au NPs	50 μM	—	Present work
**Fluorescence turn−ON method**	**LOD**	**Sensitivity**	**References**
PFS–Au NPs	25 nM	—	16
BSA–Pt–Au NCs	0.4 μM	—	48
y−CDs and Ag^+^	0.25 μM	—	49
Au @ C-dot	50 nM		50
GOx–FL Au NPs (Green FL emission)	6.8 μM	—	Present work
GOx–FL Au NPs (Red FL emission)	89 μM	—	Present work
**Electrochemical method**	**LOD**	**Sensitivity**	**References**
BCNT/GC	0.26 ± 0.01 M	25.3 ± 1.2 nA/mM	51
Au NPs/SG –PEDOT/GC Electrode	0.02 μM	—	52
OMC/GC Electrode	2.0 nM	23.6 μA/mM	53
Pt/CNTs/graphite	0.3 μM	—	54
FePc–Au NPs/GP	0.27 μM	11.24 mA/mM	55
GOx–FL Au NPs/GC Electrode	1.5 μM	0.037 μA/mM	Present work

Ag NPRs–Silver Nanoprisms.

CEQC–Carboxyl ethyl quaternized cellulose.

CMC–Carboxymethyl Cellulose.

FSN–Fluorosurfactant.

SG−Hg^2+^− MSD–Sybre Green- Mercury-Specific DNA.

PMAA–Polymethyl(methacrylic acid).

AIE–Aggregation-induced emission.

BSA–Bovine serum albumin.

y−CDs–Yellow-Emissive N-doped Carbon Dots.

C−dot–Carbon Dot.

BCNT–Boron-doped carbon nanotube.

GC–Glassy Carbon.

SG−PEDOT–Poly(3,4-ethylenedioxythiophene)- sulfonated grapheme.

OMC–Ordered mesoporous carbon.

CNTs–Carbon nanotubes.

FePc–Iron phthalocyanine.
