# Supplementary material for: Multimodal Sensing Strategy Using pH Dependent Fluorescence Switchable System
Source: Sci Rep. 2016 Dec 22;6:39452. doi: 10.1038/srep39452 (PMC5177922; doi:10.1038/srep39452)
Supplement: Supplementary Information [file srep39452-s1.pdf]

<u>**Electronic Supplementary Information (ESI) File:**</u>

Multimodal Sensing Strategy Using pH Dependent Fluorescence Switchable System

A. Muthurasu[1,2] and V. Ganesh[1,2,*]

[1]*Electrodics and Electrocatalysis (EEC) Division, CSIR – Central Electrochemical Research Institute (CSIR – CECRI), Karaikudi – 630003, Tamilnadu, India.*
[2]*Academy of Scientific and Innovative Research (AcSIR), New Delhi – 110025, India.*
Tel: +91-4565-241242; Fax: +91-4565-227779.
Corresponding Author's E-mail: vganesh@cecri.res.in (or) ganelectro@gmail.com

Figures:

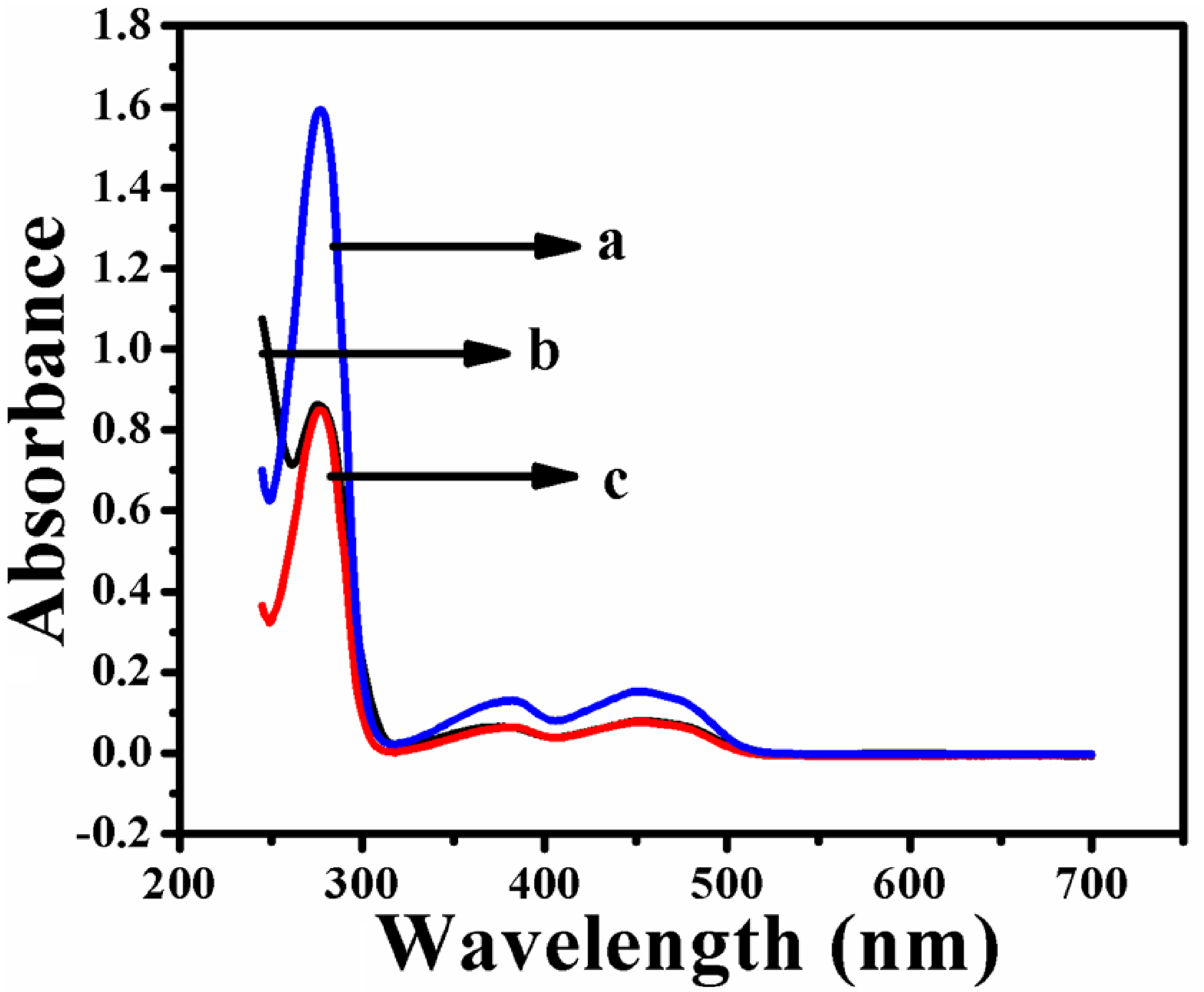

Figure S1: UV – Visible spectra of the enzyme GOx alone in neutral (pH=7) (a), acidic (pH=5) (b) and basic (pH=10) (c) conditions respectively.

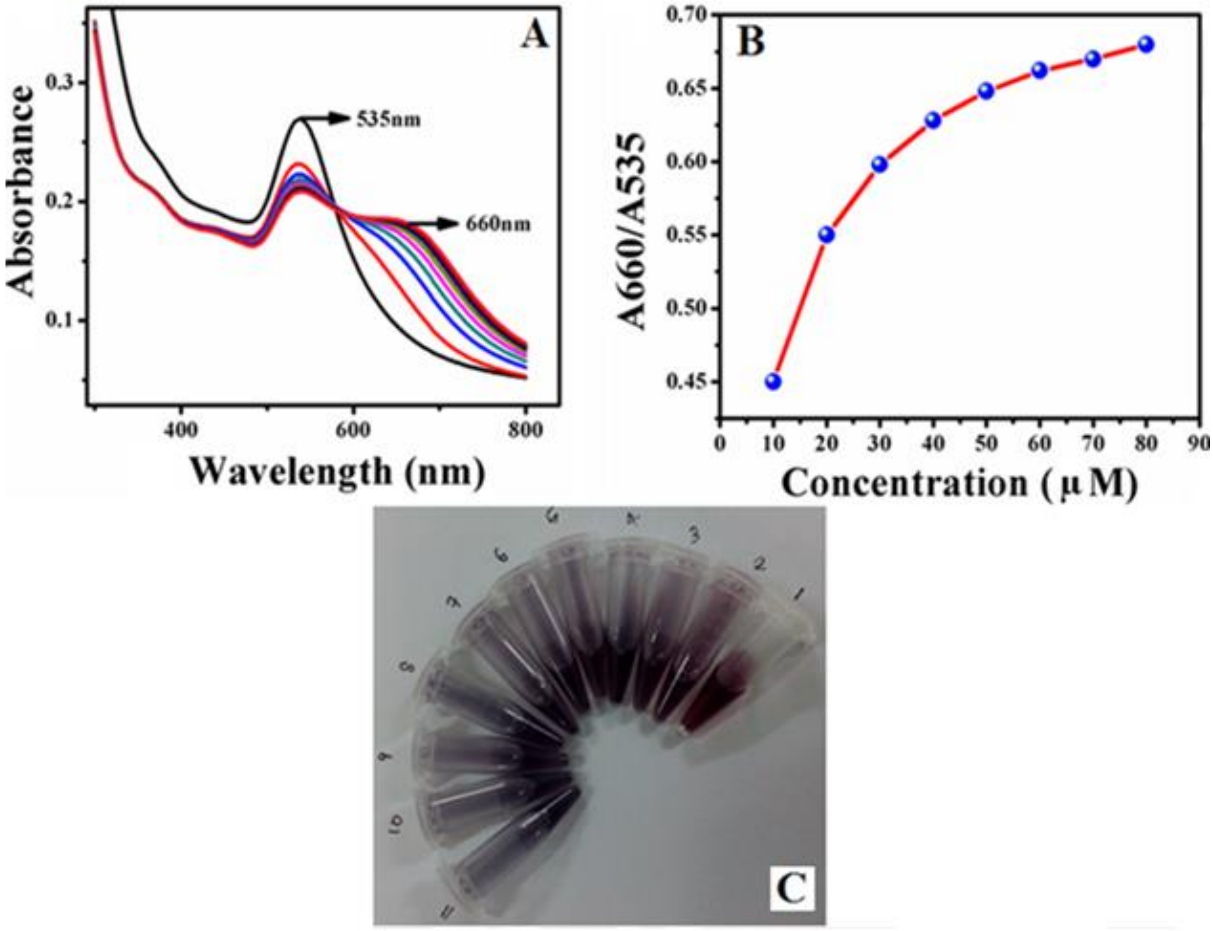

Figure S2: (A) UV – Visible spectra of GOx – FL Au NPs for the addition of various concentrations of Cys. (B) A plot of absorption ratio (A660/A535) vs. concentration of Cys. Data points are obtained from Figure A. (C) Visual photographic images highlighting the colour change from red to blue with the addition of 0 µM (blank, 1), 10 µM (2), 20 µM (3), 30 µM (4), 40 µM (5), 50 µM (6), 60 µM (7), 70 µM (7), 80 µM (8), 90 µM (9), 100 µM (10) and 110 µM (11) concentrations of Cys respectively.

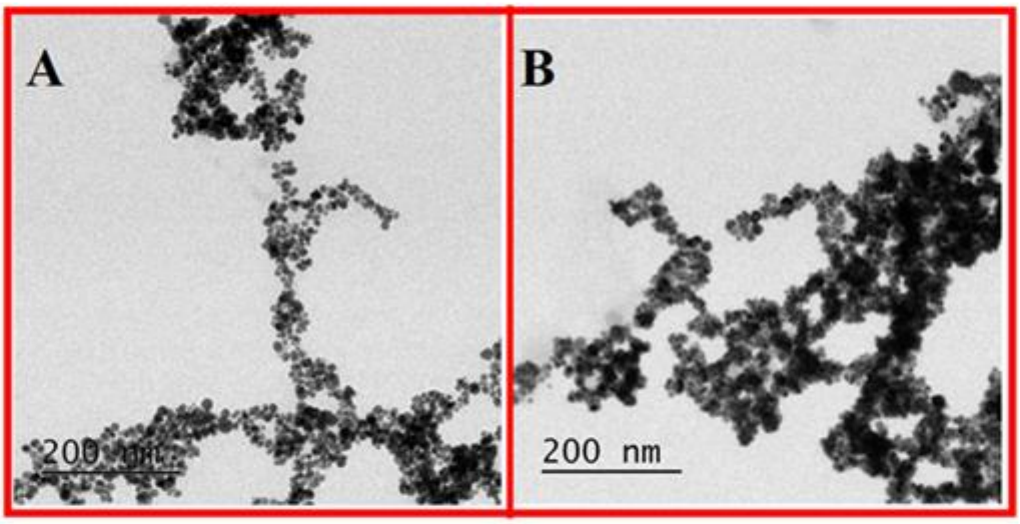

Figure S3: TEM images of GOx – FL Au NPs obtained after the addition of Cys resulting in the formation of agglomerated Au NPs. These images are recorded at two different locations.

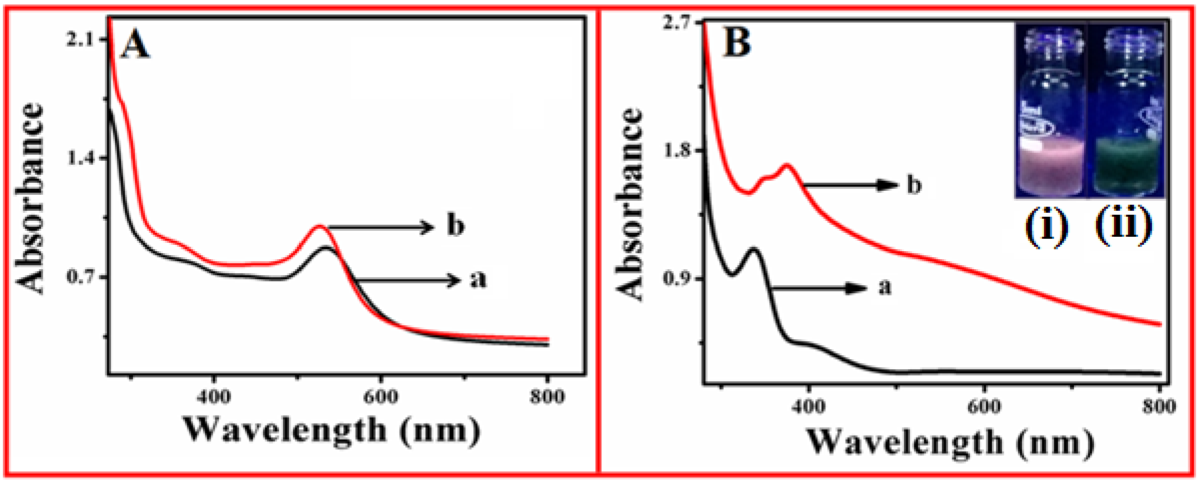

Figure S4: (A) UV – Visible absorbance spectra of GOx – FL Au NPs solution under (a) acidic (pH=5) and (b) basic (pH=10) conditions without the addition of Cys. (B) Similar UV – Visible absorbance spectra recorded using GOx – FL Au NPs solution under (a) acidic (pH=5) and (b) basic (pH=10) conditions in presence of fixed concentration of Cys. Inset shows the fluorescence emission corresponding to GOx – FL Au NPs observed under acidic (Rosy-red, i) and basic (Green colour, ii) conditions on adding Cys under exposure to UV light.

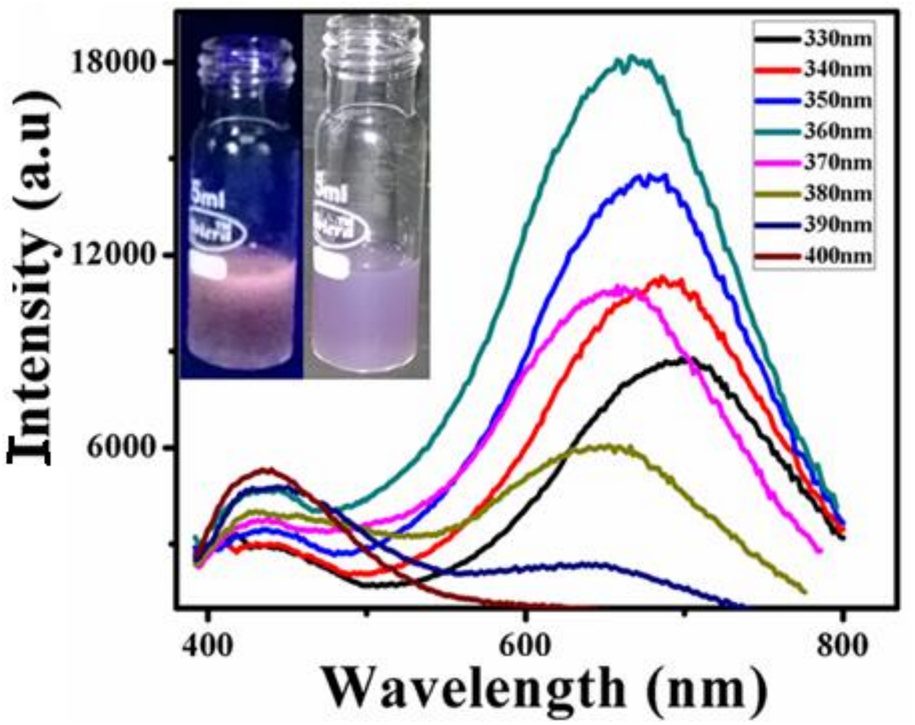

Figure S5: Fluorescence emission spectra recorded at different excitation wavelengths ranging from 330 nm to 400 nm for GOx – FL Au NPs under acidic (pH=5) condition consisting of 1mM Cys. Inset displays the photographs of GOx – FL Au NPs at pH 5 for the addition of 1mM Cys under normal visible light and after exposure to UV light (rosy-red fluorescence emission).

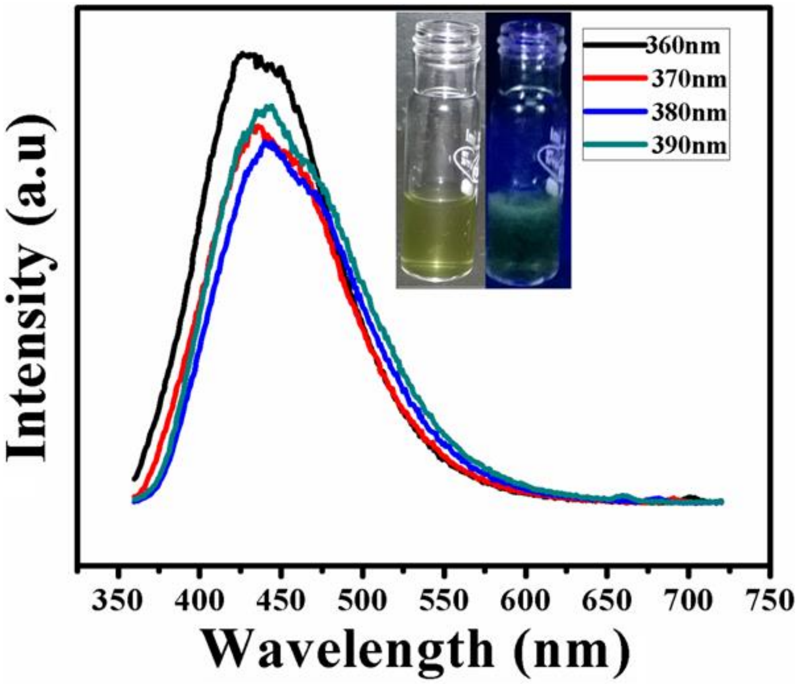

Figure S6: Fluorescence emission spectra recorded at different excitation wavelengths ranging from 360 nm to 390 nm for GOx – FL Au NPs under basic (pH=10) condition consisting of 10 μM Cys. Inset displays the photographs of GOx – FL Au NPs at pH 10 for the addition of 10 μM Cys under normal visible light and after exposure to UV light (green fluorescence emission).

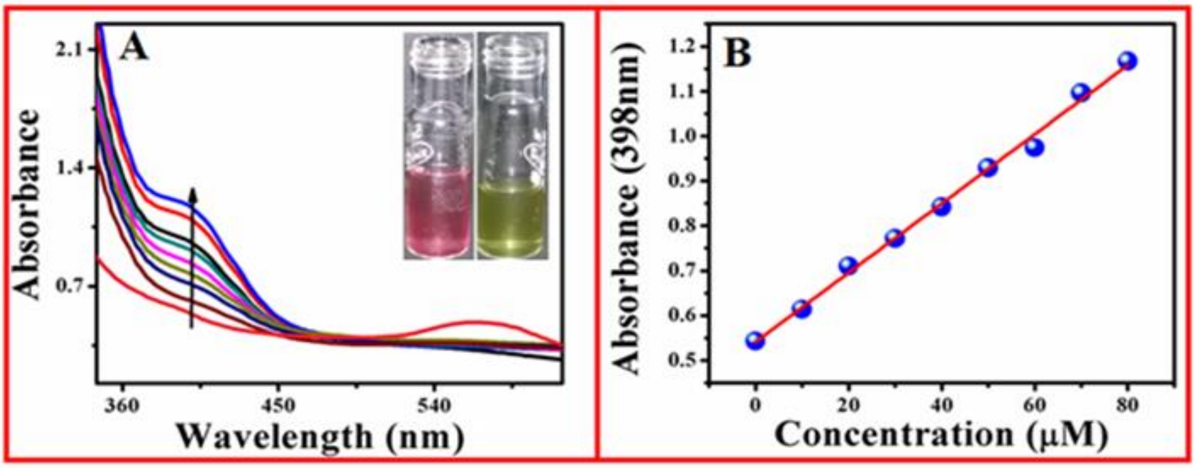

Figure S7: (A) UV – Visible absorbance spectra of GOx – FL Au NPs recorded at pH 10 for the addition of various concentrations of Cys. (B) A plot of linear variation of absorbance value measured at 398 nm vs. concentration of Cys added. Inset: Photographs of GOx – FL Au NPs at pH 10 under normal visible light before adding Cys (red colour) and after adding Cys (pale green colour).

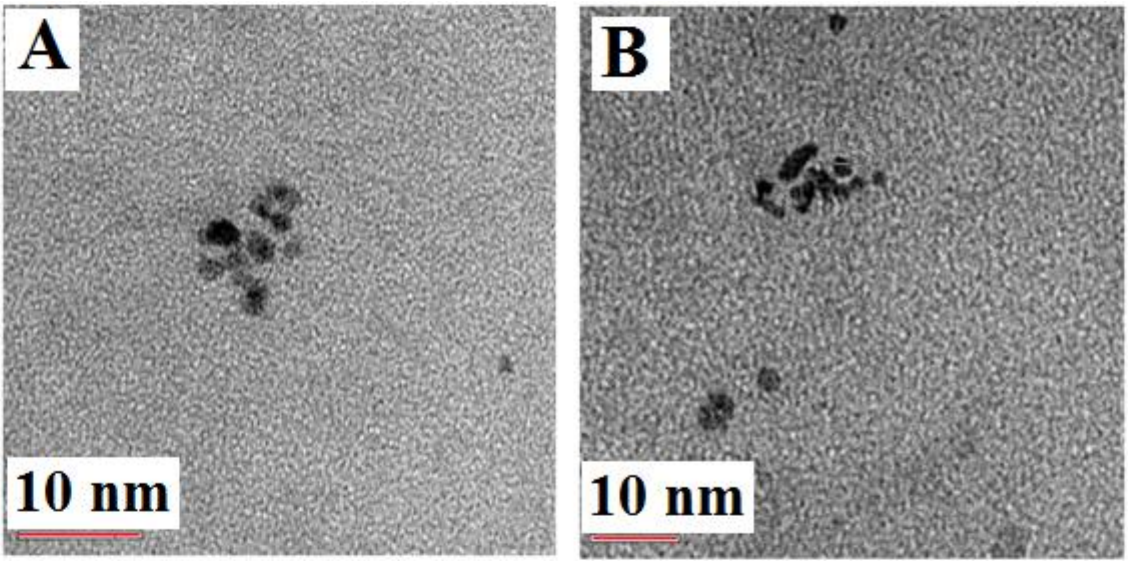

Figure S8: TEM images obtained for GOx – FL Au NPs after the addition of Cys under acidic (A, pH=5) and basic (B, pH=10) conditions.

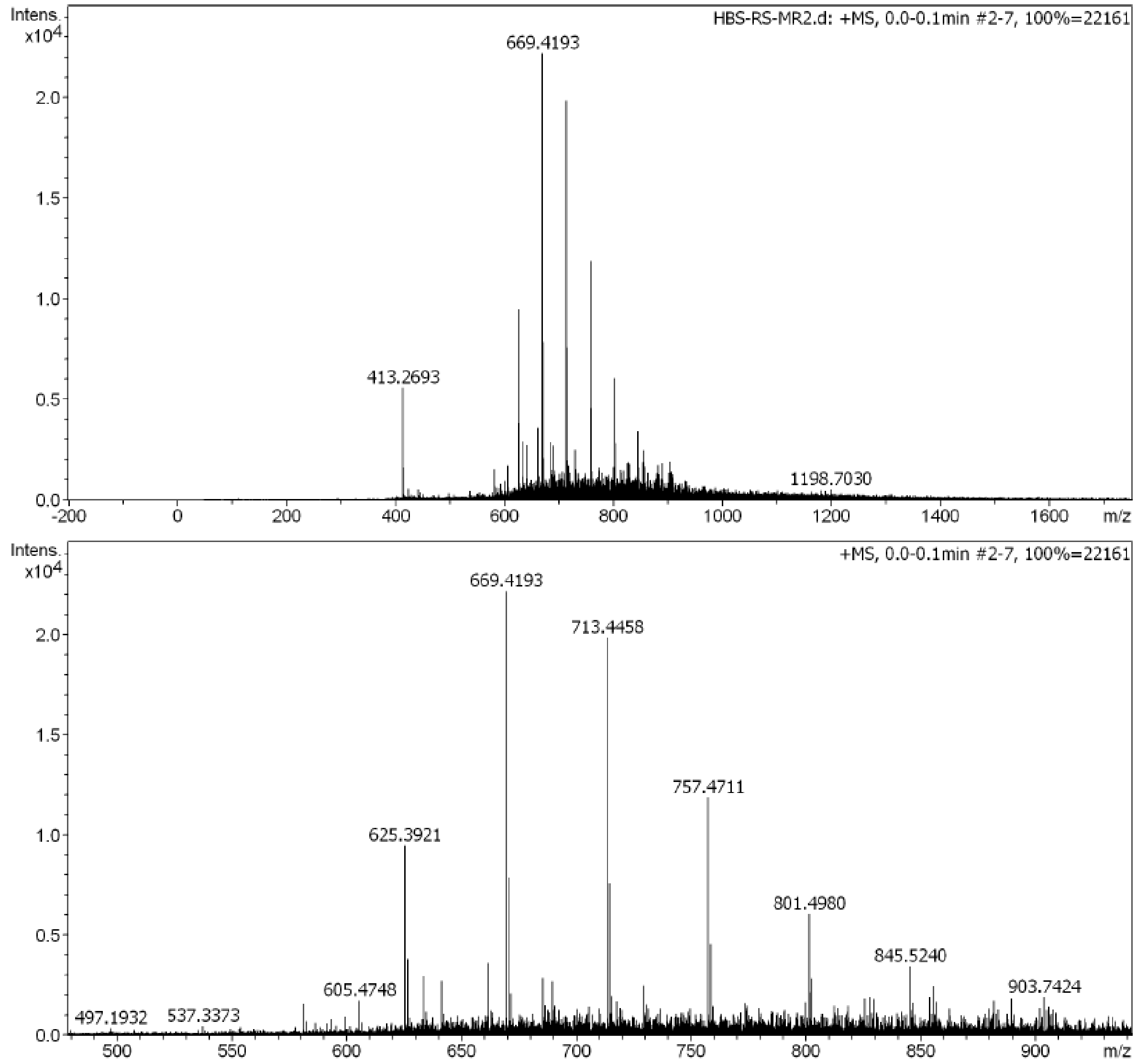

Figure S9: Mass spectroscopic (ESI-MS) data obtained for GOx – FL Au NPs after the addition of Cys under acidic (pH=5) condition and its zoomed version. Appearance of these peaks indicates the formation of Au nanoclusters primarily comprising of $Au_3(Cys)H_2O$, $Au_5(Cys)_2 2H_2O$, $Au_7(Cys)_8 8H_2O$.

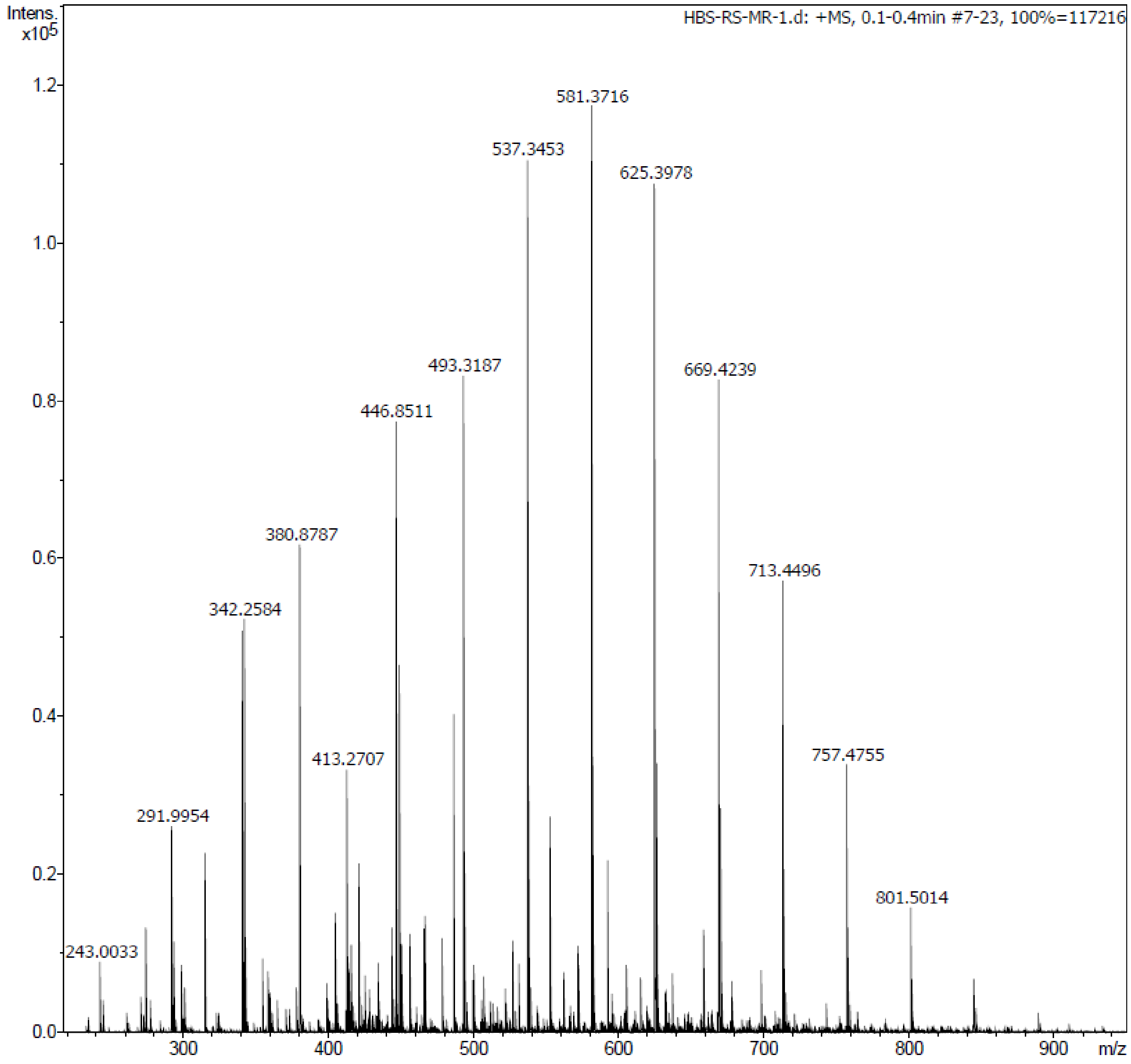

Figure S10: Mass spectroscopic data obtained for GOx – FL Au NPs after the addition of Cys under basic (pH=10) condition. Appearance of these peaks indicates the formation of Au nanoclusters consisting of $Au_2(Cys)H_2O$, $Au_3(Cys)7H_2O$, $Au_2(Cys)_22H_2O$, $Au_2(Cys)_25H_2O$, $Au_3(Cys)_3H2O$ and $Au_5(Cys)_32H_2O$.

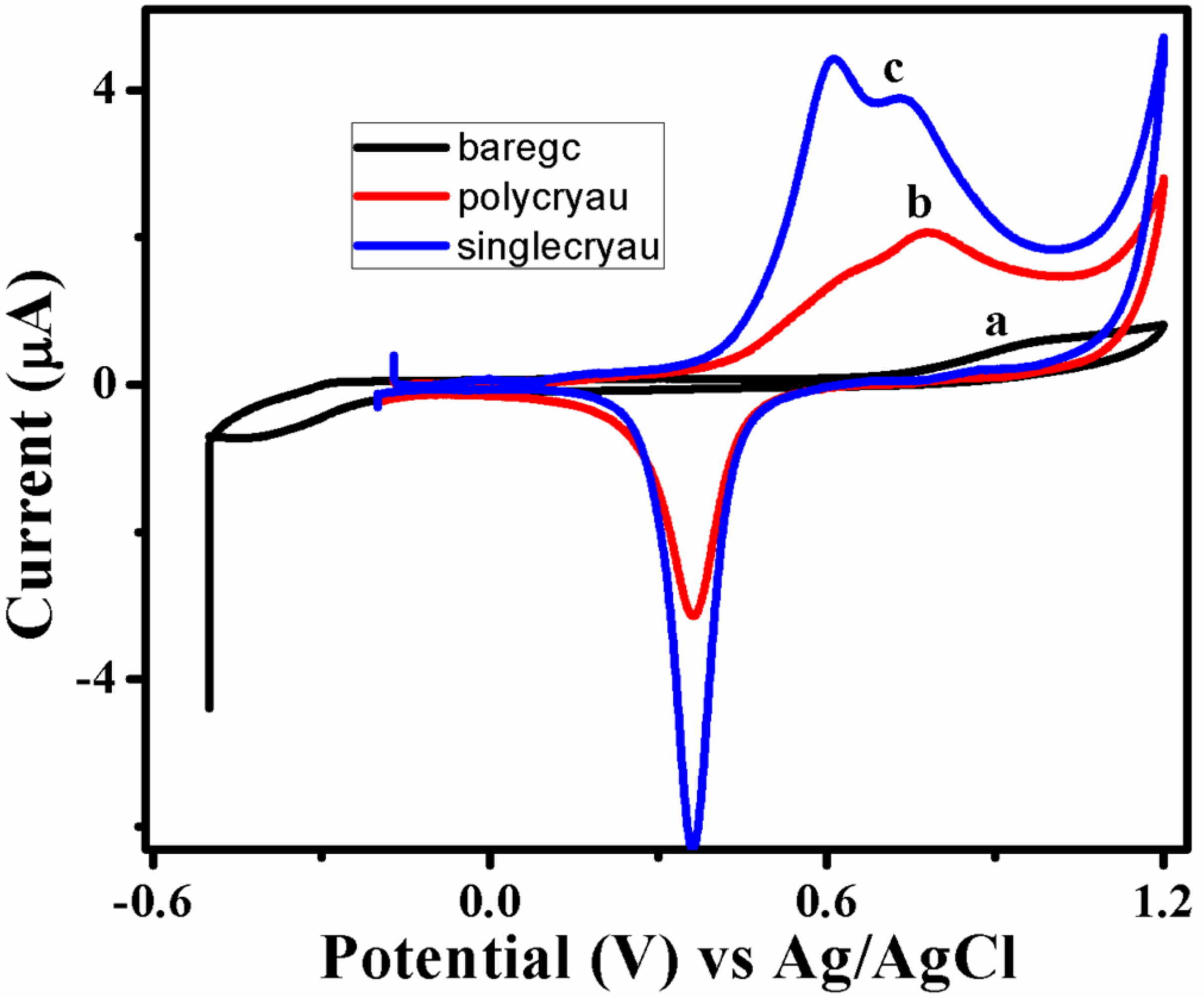

Figure S11: Cyclic voltammograms recorded in an aqueous solution of 0.1 M PBS consisting of 1.0 mM Cys at a fixed scan rate of 50 mV/s for bare GC electrode (a), bare polycrystalline Au electrode (b) and bare single crystalline Au (111) electrode (c) respectively.

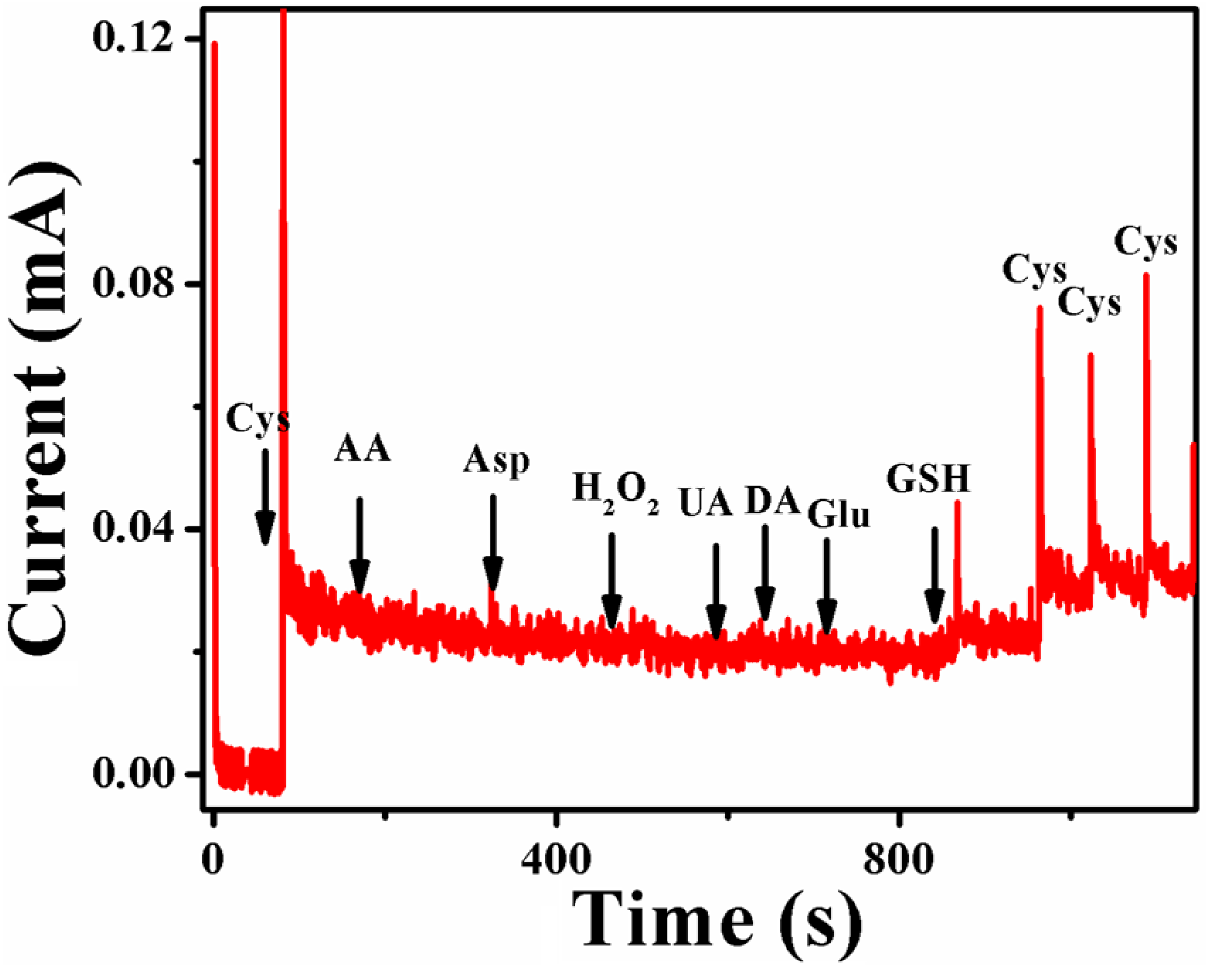

<u>Figure S12:</u> Chronoamperometric data obtained using GOx – FL Au NPs modified GC electrode under stirred condition in 0.1M PBS aqueous solution at a fixed potential of 0.75 V vs. Ag/AgCl showing the effect of various interference molecules towards electrochemical detection of Cys.
